# Daratumumab plus lenalidomide maintenance in newly diagnosed multiple myeloma after transplant: AURIGA subgroup analyses

**DOI:** 10.1038/s41408-025-01355-0

**Published:** 2025-10-06

**Authors:** Laahn Foster, Larry D. Anderson, Alfred Chung, Chakra P. Chaulagain, Erin Pettijohn, Andrew J. Cowan, Caitlin Costello, Sarah Larson, Douglas W. Sborov, Kenneth H. Shain, Rebecca Silbermann, Peter Voorhees, Maria Krevvata, Huiling Pei, Sharmila Patel, Vipin Khare, Annelore Cortoos, Robin Carson, Thomas S. Lin, Ashraf Badros

**Affiliations:** 1 Division of Hematology Oncology, University of Virginia, Charlottesville, VA USA; 2 Myeloma, Waldenstrom’s and Amyloidosis Program, Simmons Comprehensive Cancer Center, UT Southwestern Medical Center, Dallas, TX USA; 3 Department of Medicine, University of California San Francisco, San Francisco, CA USA; 4 Department of Hematology and Oncology, Myeloma and Amyloidosis Program, Cleveland Clinic Florida, Weston, FL USA; 5 Cancer and Hematology Centers of Western Michigan, Grand Rapids, MI USA; 6 Division of Medical Oncology, Fred Hutch Cancer Center/University of Washington, Seattle, WA USA; 7 Moores Cancer Center, University of California San Diego, La Jolla, CA USA; 8 Division of Hematology and Oncology, David Geffen School of Medicine at UCLA, Los Angeles, CA USA; 9 Huntsman Cancer Institute, University of Utah, Salt Lake City, UT USA; 10 Department of Malignant Hematology, H. Lee Moffitt Cancer Center, Tampa, FL USA; 11 Knight Cancer Institute, Oregon Health & Science University, Portland, OR USA; 12 Levine Cancer Institute, Atrium Health Wake Forest University School of Medicine, Charlotte, NC USA; 13 Johnson & Johnson, Spring House, PA USA; 14 Johnson & Johnson, Titusville, NJ USA; 15 Johnson & Johnson, Horsham, PA USA; 16 Greenebaum Comprehensive Cancer Center, University of Maryland, Baltimore, MD USA

**Keywords:** Drug development, Myeloma, Therapeutics

## Abstract

In the primary analysis (32.3-month median follow-up) of the randomized, phase 3 AURIGA study (NCT03901963), daratumumab-lenalidomide (D-R) maintenance significantly improved MRD-negative conversion rates and reduced the risk of disease progression or death by 47% versus R maintenance in anti-CD38 monoclonal antibody–naïve and post-transplant MRD-positive patients with newly diagnosed MM. Here, we present a post hoc analysis across relevant subgroups, including high-risk cytogenetic abnormalities (HRCAs) per original, revised, and modified International Myeloma Society (IMS) 2024 criteria. MRD-negative (10^−5^) conversion rates by 12 months of maintenance were higher for D-R versus R across cytogenetically high-risk subgroups per original (31.8% vs 6.7%), revised (43.8% vs 13.3%), and modified IMS 2024 (41.2% vs 0%) criteria and cytogenetically ultra-high–risk disease (≥2 revised HRCAs; 54.5% vs 0%). Similar trends in overall MRD-negative conversion rates were observed across subgroups. D-R demonstrated a trend towards improved PFS versus R (HR [95% CI]) in cytogenetically high-risk subgroups per original (0.60 [0.21–1.70]), revised (0.53 [0.21–1.31]), and modified IMS 2024 (0.45 [0.13–1.53]) criteria and cytogenetically ultra-high–risk disease (0.61 [0.17–2.25]). Similar outcomes were observed regardless of age or race, with no additional safety concerns among older (≥65 years) or Black patients. These data support the benefit of D-R maintenance regardless of age, race, and risk status.

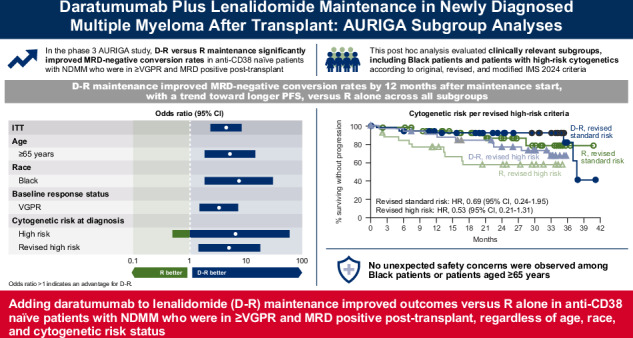

## Introduction

Daratumumab is a human IgGκ monoclonal antibody (mAb) targeting CD38 with direct on-tumor [1–4] and immunomodulatory [5–7] mechanisms of action. Due to its favorable clinical benefit [8–13], daratumumab is approved as monotherapy for patients with relapsed/refractory multiple myeloma (MM) and in combination with standard-of-care regimens for patients with relapsed/refractory or newly diagnosed MM (NDMM) [14, 15]. Moreover, daratumumab is the first anti-CD38 mAb to exhibit clinical benefit among transplant-eligible [9, 16], non-transplant [17], and transplant-ineligible patients with NDMM [8, 10] across multiple phases of therapy, including induction/consolidation and maintenance.

The randomized phase 3 AURIGA study (ClinicalTrials.gov Identifier, NCT03901963) was the first study designed to evaluate the addition of subcutaneous (SC) daratumumab to lenalidomide (D-R) maintenance in transplant-eligible patients with NDMM who were anti-CD38 mAb naïve, minimal residual disease (MRD) positive, and had achieved very good partial response or better (≥VGPR) after autologous stem cell transplant (ASCT) [18]. In the primary analysis of AURIGA (median follow-up, 32.3 months), D-R maintenance resulted in a significant increase in the MRD-negative (10^–5^) conversion rate by 12 months of maintenance versus lenalidomide (R) alone (50.5% vs 18.8%, respectively; odds ratio, 4.51 [95% confidence interval (CI), 2.37–8.57]; *P* < 0.0001) and a 47% reduction in the risk of disease progression or death (hazard ratio [HR], 0.53 [95% CI, 0.29–0.97]; *P* = 0.0361) [18].

Despite rapid advancements in the treatment of NDMM, certain patient and disease characteristics predict poor prognosis [19], including ethnicity, age, and the presence of high-risk cytogenetic abnormalities (HRCAs) [20]. For several decades, high-risk cytogenetics have been defined based upon the presence of t(4;14), t(14;16), and/or del(17p) [21]. Several years ago, this definition was revised to also include the presence of t(14;20) and/or gain/amp(1q21) as HRCAs [22]. With continued advancement in cytogenetic assessment methodologies and increased availability of clinical trial data in patients with specific HRCAs, an updated consensus definition of high-risk MM was recently presented at the 2024 International Myeloma Society (IMS) Annual Meeting, stating the single occurrence of either del(17p) with 20% of cells positive, TP53 mutation, or biallelic del(1p32) is sufficient to meet the criteria for high-risk disease [23, 24]. Co-occurrence of monoallelic del(1p32) with gain/amp(1q21) and co-occurrence of t(4;14), t(14;16), and/or t(14;20) with gain/amp(1q21) and/or monoallelic del(1p32) are also considered high-risk disease, as well as the combination of high beta-2-microglobulin (ß2M; >5.5 mg/dL) with normal creatinine (<1.2 mg/dL) [23, 24].

Clinical studies are crucial to determine the efficacy of varying treatment strategies in patients with commonly defined and newly emerging high-risk characteristics. Given that the IMS 2024 high-risk MM definition was presented very recently, few studies have reported on the clinical outcomes of patients with these specific risk factors. Therefore, we present a post hoc analysis of several clinically relevant subgroups from AURIGA, including older adults, Black patients, patients with high-risk disease per International Staging System (ISS), and patients with cytogenetically high-risk disease per the original, revised, and the recent IMS 2024 criteria (slightly modified based on data availability).

## Methods

### Patients and study design

The detailed design of the multicenter, randomized, open-label, active-controlled, phase 3 AURIGA study has been previously reported [18]. The study protocol and all amendments were approved by the institutional review board or independent ethics committee at each site, a listing of which can be found in the **Supplementary Appendix**. The study adhered to the International Council for Harmonisation’s good clinical practice guidelines and the principles of the Declaration of Helsinki, complying with all relevant regulatory and country-specific requirements. Written informed consent was obtained from all patients. The full protocol and analysis plan can be accessed at ClinicalTrials.gov.

Briefly, the study enrolled patients from 52 sites across the United States and Canada who were aged 18–79 years, had NDMM with ≥4 prior cycles of induction therapy, had received high-dose therapy and ASCT within 12 months of the start of induction, were randomized within 6 months of ASCT, were anti-CD38 mAb naïve, were MRD positive (10^–5^ by next-generation sequencing; Adaptive Biotechnologies), had achieved ≥VGPR (per International Myeloma Working Group [IMWG] 2016 criteria) post-ASCT [25], and had an Eastern Cooperative Oncology Group performance status of 0–2. Eligible patients were randomized 1:1 via an interactive web response system to receive D-R or R maintenance after stratification by cytogenetic risk per investigator’s assessment (standard risk/unknown vs high risk), as assessed by fluorescence in situ hybridization/karyotype testing, with high risk defined as the presence of ≥1 of the following HRCAs: del(17p), t(4;14), and/or t(14;16).

All patients received 10 mg of R daily on Days 1–28 of each 28-day cycle, potentially increasing to a dose of 15 mg after 3 cycles if tolerated and per investigator discretion. Patients randomized to the D-R group also received daratumumab SC (1800 mg daratumumab co-formulated with recombinant human hyaluronidase PH20 [2000 U/mL; ENHANZE^®^ drug delivery technology; Halozyme, Inc.]) weekly during Cycles 1 and 2, every 2 weeks during Cycles 3–6, and every 4 weeks from Cycle 7 thereafter. Treatment continued for a planned maximum duration of 36 cycles or until disease progression, unacceptably toxicity, or consent withdrawal.

### Subgroups

This post hoc analysis included the intent-to-treat (ITT) population, defined as all patients who were randomized to the study treatment. Patients were categorized into the following patient subgroups: race (Black or White), age (<65 years or ≥65 years), ISS disease stage at diagnosis (I, II, or III), baseline response status at study entry (VGPR or complete response or better [≥CR]), cytogenetic risk per original definition (del[17p], t[4;14], and/or t[14;16]), cytogenetic risk per revised definition (t[4;14], t[14;16], t[14;20], del[17p], and/or gain/amp[1q21]), number of HRCAs (0, 1, or, ≥2 per the revised definition), gain/amp(1q21), isolated gain/amp(1q21), and risk per modified IMS 2024 criteria. In AURIGA, data were not collected on TP53 mutation, differentiation between monoallelic versus biallelic del(1p32), or ß2M and creatinine levels at the time of MM diagnosis; hence, the IMS 2024 high-risk definition was modified to the following: ≥20% del(17p); del(1p32) co-occurring with gain/amp(1q21); and t(4;14), (14;16), and/or (14;20) co-occurring with gain/amp(1q21) and/or del(1p32). Risk assessment used mutation data collected at diagnosis and was based on local laboratory results. Subgroup definitions can be found in Supplementary Table 1.

### Endpoints and assessments

The primary endpoint of AURIGA was MRD-negative conversion rate from baseline to 12 months of maintenance, defined as the proportion of patients who converted to MRD-negative status (10^–5^) by 12 months after the initiation of maintenance treatment and prior to progressive disease or initiation of subsequent antimyeloma therapy, and has been previously published [18]. Endpoints explored in this post hoc analysis of clinically relevant subgroups include MRD-negative conversion rate (10^–5^) by 12 months of maintenance, overall MRD-negative conversion rate, ≥CR rate, and progression-free survival (PFS).

Per protocol, MRD was assessed by next-generation sequencing of bone marrow aspirate samples by central laboratory (clonoSEQ^®^; Adaptive Biotechnologies) at a minimum sensitivity threshold of 10^–5^. Bone marrow samples were collected at screening and after 12, 18, 24, and 36 months of maintenance within an accepted ± 30-day window of the scheduled visit. Response and disease progression were assessed with a validated computerized algorithm in accordance with IMWG 2016 response criteria [25].

### Statistical analysis

This post hoc analysis was conducted at the time of the primary analysis, which was conducted after all randomized patients had completed 12 months of maintenance, had disease progression, died, or discontinued study treatment. Sample size justification and additional statistical methods have been previously described [18] and are available at ClinicalTrials.gov.

The MRD-negative conversion rate by 12 months of study treatment and the overall MRD-negative conversion rate were evaluated between treatment groups using the Mantel-Haenszel estimate of the common odds ratio for stratified (ITT population) and unstratified tables (subgroups), with 2-sided 95% CIs. PFS was assessed using the Kaplan-Meier method, with HRs and 95% CIs calculated from a Cox proportional hazards model and treatment as the sole explanatory variable.

## Results

### Patients and treatment

A total of 200 patients were enrolled and randomized to receive either D-R (*n* = 99) or R (*n* = 101) maintenance. Summary of treatment disposition has been previously published [18]. Baseline demographic and disease characteristics were generally well balanced across treatment groups and have been previously published [18]; 22% of patients were Black (Supplementary Table 2). An imbalance between treatment groups in the original high-risk cytogenetic criteria (D-R, *n* = 22; R, *n* = 15) was observed, primarily due to a higher number of D-R patients having del(17p). This was driven by investigators mixing cytogenetic risk assessments at the time of randomization with assessments made based on cytogenetic data either at screening or MM diagnosis. Per the revised high-risk cytogenetic criteria, the 2 treatment groups had a similar number of patients with 1 HRCA (D-R, *n* = 21; R, *n* = 20) and ≥2 HRCAs (*n* = 11; *n* = 10), with a slight imbalance noted for isolated gain/amp(1q21) (*n* = 10; *n* = 15). For modified IMS 2024 high-risk criteria, an imbalance was observed between treatment groups, with a higher number of patients having high-risk disease in the D-R group (D-R, *n* = 17; R, *n* = 8), primarily due to the higher frequency of del(17p) (≥20% of cells positive; *n* = 10; *n* = 2), as well as an imbalance in the co-occurrence of del(1p32) with gain/amp(1q21) (*n* = 4; *n* = 0).

### Subgroup analysis of MRD-negative (10^–5^) conversion rate

**Fig. 1:**
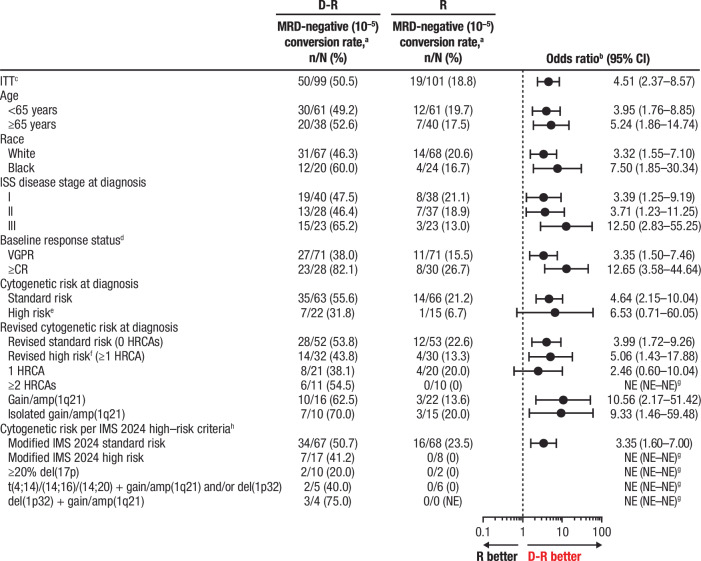
Subgroup analysis of MRD-negative (10^–5^) conversion rate by 12 months of maintenance.^a^ MRD minimal residual disease, D-R daratumumab/lenalidomide, R lenalidomide, CI confidence interval, ITT intent-to-treat, ISS International Staging System, VGPR very good partial response, CR complete response, HRCA high-risk cytogenetic abnormality, NE not estimable, IMS International Myeloma Society. ^a^Defined as the proportion of patients who were MRD positive at baseline and achieved MRD-negative status (at 10^–5^) by next-generation sequencing by 12 months after maintenance treatment and prior to progressive disease or subsequent antimyeloma therapy. ^b^Mantel-Haenszel estimate of the common odds ratio for stratified tables was used for the ITT population; Mantel-Haenszel estimate of the common odds ratio for unstratified tables was used for subgroups. An odds ratio >1 indicates an advantage for D-R maintenance. ^c^ITT population is defined as all patients who were randomized to treatment. ^d^Response status upon entering the study as assessed by International Myeloma Working Group 2016 criteria. ^e^High-risk cytogenetics per the original definition are defined as ≥1 abnormality including del(17p), t(4;14), and/or t(14;16). ^f^Revised high-risk cytogenetics per the revised definition are defined as ≥1 abnormality including del(17p), t(4;14), t(14;16), t(14;20), and/or gain/amp(1q21). ^g^Not evaluable because no patient in the R group had MRD-negative conversion. ^h^High risk per the modified IMS 2024 criteria is defined as the presence of ≥20% del(17p); del(1p32) co-occurring with gain/amp(1q21); or t(4;14), t(14;16), and/or t(14;20) co-occurring with gain/amp(1q21) and/or del(1p32). In the AURIGA study, data were not available on TP53 mutations, beta-2-microglobulin and creatinine levels at the time of multiple myeloma diagnosis, and differentiation between monoallelic versus biallelic del(1p32).

At the time of this analysis, D-R improved MRD-negative (10^–5^) conversion rate by 12 months of maintenance (primary endpoint) versus R across all subgroups explored, suggesting a benefit with D-R regardless of race and age (Fig. 1). D-R was also associated with higher rates of MRD-negative (10^–5^) conversion by 12 months of maintenance across all definitions of cytogenetically high-risk disease, including per original (D-R, 31.8% [7/22]; R, 6.7% [1/15]), revised (43.8% [14/32]; 13.3% [4/30]), and modified IMS 2024 (41.2% [7/17]; 0% [0/8]) criteria. Of emphasis, among the modified IMS 2024 high-risk subgroups, no MRD-negative conversions were observed in the R group, whereas MRD-negative conversion rates for these subgroups ranged from 20%–75% with D-R. No patient with cytogenetically ultra-high–risk disease (≥2 revised HRCAs) in the R group achieved MRD-negative (10^–5^) conversion, whereas 54.5% (6/11) of patients in the D-R group achieved MRD-negative (10^–5^) conversion in this difficult-to-treat population.

D-R maintenance was also associated with higher rates of overall (any time point in study after randomization) MRD-negative (10^–5^) conversion versus R maintenance across all subgroups, including those with ≥2 HRCAs (D-R, 63.6% [7/11]; R, 0% [0/10]) and in the modified IMS 2024 high-risk subgroups (D-R, 40%–75%; R, 0%; Supplementary Fig. 1).

### Subgroup analysis of CR rate

In an analysis of best response on study, or the achievement of ≥CR rate while on study treatment, treatment with D-R maintenance demonstrated a trend towards improvement of study entry response versus R maintenance across the majority of subgroups (Supplementary Fig. 2). This trend was also maintained among patients with various definitions of cytogenetically high-risk disease, including per original (D-R, 81.8% [18/22]; R, 53.3% [8/15]), revised (87.5% [28/32]; 50.0% [15/30]), and modified IMS 2024 (76.5% [13/17]; 37.5% [3/8]) criteria. Among patients who were in VGPR at study entry, the addition of daratumumab led to a greater proportion of patients achieving ≥CR versus R maintenance alone (D-R, 66.2% [47/71]; R, 45.1% [32/71]).

### Subgroup analysis of PFS

**Fig. 2:**
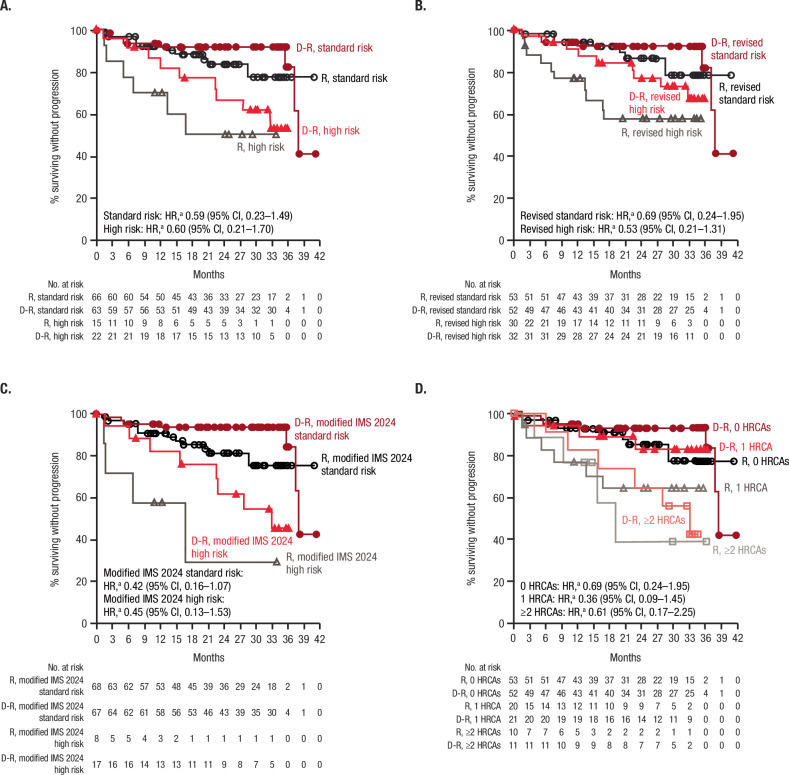
Subgroup analysis of PFS by cytogenetic risk. **A** PFS by cytogenetic risk per original criteria. High-risk cytogenetics per the original definition are defined as ≥1 abnormality including del(17p), t(4;14), and/or t(14;16). **B** PFS by cytogenetic risk per revised criteria. Revised high-risk cytogenetics per the revised definition are defined as ≥1 abnormality including del(17p), t(4;14), t(14;16), t(14;20), and/or gain/amp(1q21). **C** PFS by cytogenetic risk per modified IMS 2024 criteria. High risk per the modified IMS 2024 criteria is defined as the presence of ≥20% del(17p); del(1p32) co-occurring with gain/amp(1q21); or t(4;14), t(14;16), and/or t(14;20) co-occurring with gain/amp(1q21) and/or del(1p32). In the AURIGA study, data were not available on TP53 mutations, beta-2-microglobulin and creatinine levels at the time of multiple myeloma diagnosis, and differentiation between monoallelic versus biallelic del(1p32). **D** PFS by the number of HRCAs, defined as the number of abnormalities from del(17p), t(4;14), t(14;16), t(14;20), and/or gain/amp(1q21). Cytogenetic results at diagnosis (based on clinical report form collected data from local labs) were used in the analysis. PFS, progression-free survival; IMS, International Myeloma Society; HRCA, high-risk cytogenetic abnormality; D-R, daratumumab/lenalidomide; R, lenalidomide; HR, hazard ratio; CI, confidence interval. ^a^HR and 95% CI from a Cox proportional hazards model with treatment as the sole explanatory variable. An HR <1 indicates an advantage for D-R maintenance.

At a median follow-up of 32.3 months, PFS favored D-R versus R maintenance across all subgroups, suggesting a trend in PFS benefit with D-R regardless of age, race, disease stage, or various definitions of cytogenetic risk status (Supplementary Fig. 3). PFS HR point estimates were consistently <1 among patients with cytogenetically high-risk disease per the original (HR, 0.60 [95% CI, 0.21–1.70]; Fig. 2A) and revised (HR, 0.53 [95% CI, 0.21–1.31]) criteria (Fig. 2B), indicating a trend towards improvement with D-R versus R. In patients with cytogenetically high-risk disease per the original criteria, median PFS was not reached versus 16.7 months in the D-R and R groups, respectively; per the revised criteria, PFS was not reached in either group. When looking closely at patients with cytogenetically high-risk disease per modified IMS 2024 criteria, the PFS HR point estimate was even lower (HR, 0.45 [95% CI, 0.13–1.53]; Fig. 2C), with a median PFS of 32.8 and 16.7 months in the D-R and R groups, respectively, indicating that the favorable PFS trend with the addition of daratumumab SC further improved with more recent high-risk criteria. In addition, a PFS trend favoring D-R maintenance was observed versus R alone among patients with 0 HRCAs (HR, 0.69 [95% CI, 0.24–1.95]), 1 HRCA (HR, 0.36 [95% CI, 0.09–1.45]), and cytogenetically ultra-high–risk disease (≥2 HRCAs; HR, 0.61 [95% CI, 0.17–2.25]; Fig. 2D). In patients with gain/amp(1q21) irrespective of other HRCAs, the PFS HR point estimate indicated a trend towards improvement with D-R versus R alone (HR, 0.46 [95% CI, 0.13–1.59]); however, the treatment effect could not be quantified in those with isolated gain/amp(1q21) as no PFS events were observed in the D-R group versus 4 PFS events in the R group (Supplementary Fig. 4a, b). Consistent with observations across other subgroups, a PFS trend favoring D-R versus R maintenance was seen regardless of response status at study entry; however, this trend was less pronounced for patients in VGPR at study entry (Supplementary Fig. 4c).

### Safety

The safety and tolerability profiles of D-R and R maintenance in the overall AURIGA study population have been previously published [18]. The aim of this analysis was to verify any specific safety concerns in the older patient population, as well as in Black patients. When looking at safety data by race, no additional safety concerns were observed with D-R maintenance among Black patients. Median duration of therapy among White patients was 23.7 months (D-R, 29.3 months; R, 18.9 months) and among Black patients was 25.4 months (D-R, 28.3 months; R, 25.4 months). Table 1 provides a summary of the most common treatment-emergent adverse events (TEAEs) in all patients who received ≥1 dose of study treatment, separated by White (D-R, *n* = 64; R, *n* = 65) or Black (D-R, *n* = 20; R, *n* = 24) race. The incidence of grade 3/4 TEAEs was higher with D-R versus R maintenance for both White patients (D-R, 76.6%; R, 70.8%) and Black patients (75.0%; 66.7%). The most common grade 3/4 TEAE was neutropenia, with similar incidences in White patients (D-R, 45.3%; R, 43.1%) and Black patients (50.0%; 45.8%). The incidence of grade 3/4 cytopenia was similar across treatment groups for White patients (D-R, 54.7%; R, 47.7%) and Black patients (50.0%; 50.0%). The incidence of grade 3/4 infections was higher with D-R versus R maintenance among White patients (D-R, 20.3%; R, 12.3%) but was comparable among Black patients (20.0%; 20.8%). Similarly, serious TEAEs were higher with D-R versus R maintenance among White patients (D-R, 31.3%; R, 21.5%) but comparable among Black patients (30.0%; 29.2%). TEAEs leading to treatment discontinuation were highest among White patients, with greater rates observed with D-R versus R maintenance (D-R, 21.9%; R, 10.8%), while no trend was observed among Black patients (0%; 4.2%). Deaths due to TEAEs were low (*n* = 3), which all occurred among White patients (D-R, *n* = 2 [3.1%]; R, *n* = 1 [1.5%]).

**Table 1:** Summary of safety outcomes by White and Black race.

	D-R		R	
	White (*n* = 64)	Black (*n* = 20)	White (*n* = 65)	Black (*n* = 24)
Any grade TEAEs, *n* (%)	63 (98.4)	20 (100)	64 (98.5)	24 (100)
Most common^a^				
Neutropenia^b^	40 (62.5)	14 (70.0)	41 (63.1)	16 (66.7)
Diarrhea	39 (60.9)	12 (60.0)	40 (61.5)	11 (45.8)
Fatigue	31 (48.4)	8 (40.0)	29 (44.6)	12 (50.0)
URTI	26 (40.6)	9 (45.0)	18 (27.7)	5 (20.8)
Arthralgia	23 (35.9)	4 (20.0)	24 (36.9)	10 (41.7)
Cough	21 (32.8)	10 (50.0)	20 (30.8)	12 (50.0)
Hypokalemia	21 (32.8)	9 (45.0)	19 (29.2)	15 (62.5)
Back pain	20 (31.3)	7 (35.0)	12 (18.5)	4 (16.7)
Thrombocytopenia	17 (26.6)	3 (15.0)	22 (33.8)	4 (16.7)
Nausea	16 (25.0)	8 (40.0)	18 (27.7)	8 (33.3)
Leukopenia	16 (25.0)	6 (30.0)	16 (24.6)	11 (45.8)
Rash maculo-papular	14 (21.9)	6 (30.0)	15 (23.1)	2 (8.3)
Anemia	12 (18.8)	8 (40.0)	11 (16.9)	6 (25.0)
Grade 3/4 TEAEs, *n* (%)	49 (76.6)	15 (75.0)	46 (70.8)	16 (66.7)
Most common^c^				
Neutropenia^b^	29 (45.3)	10 (50.0)	28 (43.1)	11 (45.8)
Lymphopenia	9 (14.1)	0	5 (7.7)	0
Hypokalemia	6 (9.4)	1 (5.0)	3 (4.6)	3 (12.5)
Leukopenia	5 (7.8)	3 (15.0)	4 (6.2)	2 (8.3)
Diarrhea	2 (3.1)	1 (5.0)	2 (3.1)	3 (12.5)
Fatigue	0	2 (10.0)	2 (3.1)	1 (4.2)
Grade 3/4 cytopenias, *n* (%)	35 (54.7)	10 (50.0)	31 (47.7)	12 (50.0)
Grade 3/4 infections, *n* (%)	13 (20.3)	4 (20.0)	8 (12.3)	5 (20.8)
COVID-19 events, *n* (%)				
Any grade	18 (28.1)	7 (35.0)	20 (30.8)	5 (20.8)
Grade 3/4	1 (1.6)	0	1 (1.5)	2 (8.3)
Serious TEAEs, *n* (%)	20 (31.3)	6 (30.0)	14 (21.5)	7 (29.2)
Most common^d^				
Pneumonia	4 (6.3)	0	3 (4.6)	1 (4.2)
TEAEs leading to discontinuation of any treatment component,^e^ *n* (%)	14 (21.9)	0	7 (10.8)	1 (4.2)
Death due to TEAEs, *n* (%)	2 (3.1)	0	1 (1.5)	0

*D-R* daratumumab/lenalidomide, *R* lenalidomide, *TEAE* treatment-emergent adverse event, *URTI* upper respiratory tract infection.

^a^Defined as ≥30% of patients in either treatment group or racial subgroup.

^b^Preferred term grouping.

^c^Defined as ≥10% of patients in either treatment group or racial subgroup.

^d^Defined as occurring in ≥3 patients in either treatment group or racial subgroup.

^e^Includes patients who had TEAEs with an action of “drug withdrawn” taken for ≥1 component of study treatment on the “adverse event” complete report form page.

In an analysis of safety by age, no specific safety concerns were observed with the addition of daratumumab SC to R maintenance, including among older patients. The median duration of therapy among patients aged <65 years was 25.0 months (D-R, 30.5 months; R, 23.5 months) and 24.4 months (32.7 months; 19.2 months) among patients aged ≥65 years. Table 2 provides a summary of the most common TEAEs, separated by age subgroup (<65 years: D-R, *n* = 59; R, *n* = 58; ≥65 years: D-R, *n* = 37; R, *n* = 40). The incidence of grade 3/4 TEAEs was higher for D-R versus R maintenance among patients aged <65 years (D-R, 76.3%; R, 63.8%) but comparable between treatments among patients aged ≥65 years (70.3%; 72.5%). Neutropenia was the most common grade 3/4 TEAE both in patients aged <65 years (D-R, 44.1%; R, 43.1%) and in those aged ≥65 years (51.4%; 40.0%). A similar proportion of patients aged <65 years and ≥65 years experienced grade 3/4 cytopenia within each treatment group, with a higher overall incidence reported with D-R compared with R among both the younger (D-R, 52.5%; R, 46.6%) and older patients (56.8%; 47.5%). The incidence of grade 3/4 infections was lower among patients aged <65 who received R maintenance (D-R, 18.6%; R, 10.3%) but was similar across treatment groups among patients aged ≥65 years (18.9%; 17.5%). Among patients aged <65 years, the incidence of serious TEAEs was higher with D-R versus R maintenance (D-R, 23.7%; R, 12.1%), whereas for patients aged ≥65 years, the incidence was similar (40.5%; 37.5%). The frequency of TEAEs leading to treatment discontinuation was higher with D-R versus R maintenance in both age groups (<65 years: D-R, 11.9%; R, 6.9%; ≥65 years: D-R, 18.9%; R, 10.0%). Deaths due to TEAEs were low (*n* = 3), with all deaths occurring among the older patients (D-R, *n* = 2 [5.4%]; R, *n* = 1 [2.5%]).

**Table 2:** Summary of safety outcomes by age (<65 years and ≥65 years).

	D-R		R	
	<65 years (*n* = 59)	≥65 years (*n* = 37)	<65 years (*n* = 58)	≥65 years (*n* = 40)
Any grade TEAEs, *n* (%)	58 (98.3)	37 (100)	58 (100)	39 (97.5)
Most common^a^				
Neutropenia^b^	40 (67.8)	22 (59.5)	36 (62.1)	24 (60.0)
Diarrhea	33 (55.9)	26 (70.3)	31 (53.4)	23 (57.5)
Fatigue	31 (52.5)	13 (35.1)	25 (43.1)	21 (52.5)
URTI	26 (44.1)	14 (37.8)	12 (20.7)	14 (35.0)
Cough	23 (39.0)	14 (37.8)	26 (44.8)	10 (25.0)
Back pain	22 (37.3)	9 (24.3)	12 (20.7)	8 (20.0)
Hypokalemia	20 (33.9)	13 (35.1)	24 (41.4)	12 (30.0)
Arthralgia	20 (33.9)	12 (32.4)	20 (34.5)	16 (40.0)
Leukopenia	19 (32.2)	6 (16.2)	17 (29.3)	12 (30.0)
Nasal congestion	19 (32.2)	6 (16.2)	14 (24.1)	5 (12.5)
Headache	18 (30.5)	6 (16.2)	9 (15.5)	8 (20.0)
Nausea	14 (23.7)	12 (32.4)	17 (29.3)	9 (22.5)
Thrombocytopenia	13 (22.0)	10 (27.0)	11 (19.0)	17 (42.5)
Grade 3/4 TEAEs, *n* (%)	45 (76.3)	26 (70.3)	37 (63.8)	29 (72.5)
Most common^c^				
Neutropenia^b^	26 (44.1)	19 (51.4)	25 (43.1)	16 (40.0)
Lymphopenia	7 (11.9)	3 (8.1)	3 (5.2)	2 (5.0)
Hypertension	6 (10.2)	1 (2.7)	3 (5.2)	1 (2.5)
Leukopenia	6 (10.2)	3 (8.1)	2 (3.4)	4 (10.0)
Hypokalemia	4 (6.8)	3 (8.1)	2 (3.4)	4 (10.0)
Pneumonia	1 (1.7)	4 (10.8)	1 (1.7)	3 (7.5)
Grade 3/4 cytopenias, *n* (%)	31 (52.5)	21 (56.8)	27 (46.6)	19 (47.5)
Grade 3/4 infections, *n* (%)	11 (18.6)	7 (18.9)	6 (10.3)	7 (17.5)
COVID-19 events, *n* (%)				
Any grade	19 (32.2)	9 (24.3)	22 (37.9)	7 (17.5)
Grade 3/4	1 (1.7)	0	3 (5.2)	0
Serious TEAEs, *n* (%)	14 (23.7)	15 (40.5)	7 (12.1)	15 (37.5)
Most common^d^				
Pneumonia	1 (1.7)	3 (8.1)	1 (1.7)	3 (7.5)
TEAEs leading to discontinuation of any treatment component,^e^ *n* (%)	7 (11.9)	7 (18.9)	4 (6.9)	4 (10.0)
Death due to TEAEs, *n* (%)	0	2 (5.4)	0	1 (2.5)

*D-R* daratumumab/lenalidomide, *R* lenalidomide, *TEAE* treatment-emergent adverse event, *URTI* upper respiratory tract infection.

^a^Defined as ≥30% of patients in either treatment group or age subgroup.

^b^Preferred term grouping.

^c^Defined as ≥10% of patients in either treatment group or age subgroup.

^d^Defined as occurring in ≥3 patients in either treatment group or age subgroup.

^e^Includes patients who had TEAEs with an action of “drug withdrawn” taken for ≥1 component of study treatment on the “adverse event” complete report form page.

## Discussion

Despite treatment advancements in NDMM, certain patient subgroups continue to have inferior outcomes [19]. Here, we report data from a post hoc analysis of the phase 3 AURIGA study in patient subgroups with high unmet medical need, including older and Black patients and those with cytogenetically high-risk disease according to established and recent criteria. In all subgroups explored, the addition of daratumumab SC to R maintenance led to an improved MRD-negative (10^–5^) conversion rate from baseline to 12 months of maintenance and overall MRD-negative conversion rate, regardless of race, age, and risk status. In addition, favorable trends in response from baseline and PFS were also observed with D-R maintenance, with no unexpected safety concerns among Black patients or patients aged ≥65 years.

Among patients with NDMM, HRCAs have been shown to confer suboptimal outcomes and are highly prognostic; therefore, these high-risk patients constitute a group with high unmet medical need. The original definition of cytogenetically high-risk disease included the presence of abnormalities such as t(4;14), t(14;16), and/or del(17p) [21]. A subsequent “revised” definition encompassed additional factors, including t(14;20) and gain/amp(1q) abnormalities [22]. Most recently, an updated IMWG consensus definition of cytogenetically high-risk MM was presented at the 2024 annual IMS meeting [23, 24]. This new definition includes additional genomic factors, including the presence of TP53 mutation and biallelic del(1p32), both associated with worse overall survival [26, 27]. A clinical threshold of ≥20% for del(17p); the co-occurrence of monoallelic del(1p32) with gain/amp(1q21); and the co-occurrence of t(4;14), t(14;16), and/or t(14;20) with gain/amp(1q21) and/or monoallelic del(1p32) were also included as high-risk criteria [24]. While this updated definition aims to enhance the use of risk-stratified therapeutic approaches in clinical practice and to inform the design of clinical studies focused on high-risk MM, its recent introduction means that only a few studies have examined the efficacy of treatments for patients with these specific characteristics.

As reported here, the use of D-R maintenance led to an improvement versus R maintenance in MRD-negative (10^–5^) conversion rate by 12 months of maintenance across all subgroups, including those with cytogenetically high-risk disease per original (31.8% vs 6.7%), revised (43.8% vs 13.3%), and modified IMS 2024 high-risk (41.2% vs 0%) definitions. Similar benefits in favor of D-R maintenance were observed among the varying IMS 2024 high-risk subgroups, including those with ≥20% del(17p) (20.0% vs 0%); t(4;14), (14;16), and/or (14;20) co-occurring with gain/amp(1q21) and/or del(1p32) (40.0% vs 0%); and del(1p32) co-occurring with gain/amp(1q21) (75.0% vs 0%). Furthermore, among patients with ultra-high–risk cytogenetics (≥2 revised HRCAs), a patient population with high unmet medical need and treatment challenges, 54.5% of patients achieved MRD-negative (10^–5^) conversion by 12 months of maintenance with D-R versus 0% of patients with R maintenance alone, with similar trends observed for overall MRD-negative (10^–5^) conversion. Given that achieving MRD negativity is associated with prolonged PFS and overall survival [28], the demonstrated benefit of adding daratumumab SC to standard maintenance therapy in patients with high-risk, and especially ultra-high–risk, MM is promising. Another interesting observation includes the trend towards PFS improvement in patients with ≥CR at study entry, particularly among patients treated with D-R maintenance. This may indicate the importance of using optimal induction therapy (eg, quadruplet therapy) to obtain deep, early responses. Both optimal induction therapy and optimal maintenance therapy are needed to maximize patient outcomes in NDMM.

While cross-study comparisons should be done with caution, data from this post hoc analysis corroborate those observed in other clinical studies of transplant-eligible patients with NDMM. In a post hoc analysis of the phase 2 GRIFFIN study completed at the time of final analysis (median follow-up, 49.6 months), the addition of daratumumab SC to both bortezomib/lenalidomide/dexamethasone (D-VRd) induction/consolidation and R maintenance showed a trend towards the improvement of clinical outcomes versus VRd induction/consolidation and R maintenance among transplant-eligible patients with NDMM, including those with varying high-risk characteristics [29]. For instance, among patients with revised cytogenetically high-risk disease, a higher proportion of patients achieved MRD negativity (10^–5^) with D-VRd versus VRd (54.8% [23/42] vs 32.4% [12/37], respectively), and median PFS was not reached versus 47.9 months, respectively, thus favoring D-VRd (HR, 0.38 [95% CI, 0.14–1.01]) [29]. Similarly, in the phase 3 PERSEUS study, D-VRd induction/consolidation followed by D-R maintenance led to improved MRD-negative (10^–5^) rate and PFS across clinically relevant subgroups versus VRd followed by R maintenance [9]. Among patients with cytogenetically high-risk disease per the revised definition, the overall MRD-negative (10^–5^) rate was greater with D-VRd versus VRd (73.1% [95/130] vs 49.3% [73/148], respectively), and PFS similarly favored D-VRd (HR, 0.53 [95% CI, 0.35–0.81]) [30]. Furthermore, in an analysis of the ITT maintenance population from the phase 3 CASSIOPEIA study, PFS consistently favored daratumumab maintenance versus observation alone across all clinically relevant subgroups, including patients with cytogenetically high-risk disease (t[4;14] and/or del[17p]) for whom median PFS was not estimable versus 27.2 months in the daratumumab and observation groups, respectively (HR, 0.39 [95% CI, 0.25–0.63]) [13]. Collectively, these data support the benefit of adding daratumumab SC to standard-of-care maintenance treatment in clinically relevant subgroups of patients with transplant-eligible NDMM, including those with cytogenetically high-risk disease. The phase 3 CEPHEUS and IMROZ studies, which evaluated anti-CD38 mAb–based quadruplet regimens versus non–anti-CD38 mAb–based triplet regimens, demonstrated PFS HRs in transplant-ineligible and transplant-deferred patients with cytogenetically high-risk disease of 0.88 (95% CI, 0.42–1.84) [17] and 0.97 (95% CI, 0.48–1.96) [31], respectively. These results should be interpreted with caution given the limited sample size of the cytogenetically high-risk subgroups, although the PFS HRs for CD38 mAb–based quadruplet regimens in these subgroups are less favorable than the PFS HRs in all patients. Nevertheless, per data reported from the phase 3 MAIA study, favorable trends for PFS (median follow-up, 64.5 months; HR, 0.57 [95% CI, 0.34–0.96]) [32] and overall survival (median follow-up, 89.3 months; HR, 0.65 [95% CI, 0.40–1.06]) [33] were observed in transplant-ineligible patients with cytogenetically high-risk NDMM following daratumumab plus lenalidomide/dexamethasone versus lenalidomide/dexamethasone alone.

Evidence indicates that the addition of daratumumab confers benefit not only in high-risk but also standard-risk patients. The addition of daratumumab improved MRD-negative rates among patients with revised cytogenetically standard-risk disease in the AURIGA (D-R, 53.8%; R, 22.6%), GRIFFIN (D-VRd, 75.0%; VRd, 31.7%), and PERSEUS (D-VRd, 75.3%; VRd, 47.3%) studies, leading to a trend in PFS improvement (AURIGA, HR 0.69, [95% CI, 0.24–1.95]; GRIFFIN, HR 0.39 [95% CI, 0.10–1.51]; PERSEUS, HR 0.29 [95% CI, 0.15–0.56]) [18, 29, 30]. When considering the new IMS 2024 criteria, patients in AURIGA with standard-risk disease per modified IMS 2024 criteria also exhibited favorable MRD and PFS trends with the addition of daratumumab SC to R maintenance. Overall, the clinical benefit of daratumumab SC was evident in both high-risk and standard-risk disease, supporting its role as a versatile treatment option for all transplant-eligible patients with NDMM, rather than being reserved solely for patients with high-risk disease.

No specific safety concerns were identified in Black or older patients, corroborating observations from the GRIFFIN study [29, 34]. Per the AURIGA study, no increase in the incidences of grade 3/4 infection or cytopenia was observed in younger compared with older patients, nor when comparing treatment groups. In an analysis of Black patients from GRIFFIN, a greater proportion of patients experienced a higher frequency of TEAEs leading to discontinuation of ≥1 component of the study regimen (D-VRd, 64.3%; VRd, 38.9%) compared with White patients (28.9%; 25.7%); this higher frequency of TEAEs leading to discontinuation in Black patients was primarily driven by neuropathy-related events (D-VRd, 28.6%; VRd, 22.2), leading to the discontinuation of bortezomib [34]. As reported in the current analysis of AURIGA, only 4.2% of Black patients experienced TEAEs that led to discontinuation of ≥1 component of the study regimen, all of which were in the R group. Given that AURIGA enrolled a notable proportion of Black patients (22%), findings from this post hoc analysis support the safety of daratumumab SC in this patient population, which is often underrepresented in clinical studies.

This post hoc analysis has some limitations. Firstly, while subgroups were generally well balanced, a few subgroups had relatively low sample sizes, thus limiting the robustness of the observed data and the strength of inferences made. Secondly, in the AURIGA study, data were not collected for TP53 mutation, differentiation between monoallelic versus biallelic del(1p32), and ß2M or creatinine levels at the time of MM diagnosis prior to induction therapy; therefore, the impact of D-R maintenance across all IMS 2024 high-risk subgroups could not be analyzed. Given these limitations, larger phase 3 clinical studies with greater sample sizes aiming to specifically enroll patients with high-risk NDMM are needed to help draw more definitive conclusions regarding these new high-risk stratification categories. Finally, it should be noted that all patients enrolled in the AURIGA study were anti-CD38 mAb naïve, so our findings may not be applicable to patients who have received anti-CD38 mAb–based induction/consolidation treatment, which is currently considered standard of care. The phase 3 PERSEUS study, which evaluated the addition of daratumumab to VRd induction/consolidation and R maintenance, demonstrated a deepening of MRD negativity over time during D-R maintenance, suggesting a benefit in patients with prior daratumumab exposure; however, PERSEUS did not include a second randomization prior to maintenance to more specifically evaluate the role of daratumumab-based maintenance [35]. Therefore, the added value of anti-CD38 mAb treatment during maintenance after receiving anti-CD38 mAb–based induction/consolidation remains unclear; however, additional trials such as DRAMMATIC (SWOG S1803) [36] and GMMG-HD7 [37] will provide insight into this important question in the near future.

In summary, results of this post hoc analysis of AURIGA showed the consistent ability of D-R maintenance compared with standard-of-care R maintenance alone to improve MRD-negative (10^–5^) conversion rate, depth of response, and a trend towards PFS improvement among anti-CD38 mAb–naïve patients with NDMM who were MRD positive post-ASCT with either high-risk or standard-risk disease. Additionally, no unexpected safety concerns were observed among patients aged ≥65 years or Black patients. This study supports the addition of daratumumab SC to R maintenance in all transplant-eligible patients with NDMM regardless of age, race, and cytogenetic risk status; however, additional studies with larger sample sizes are needed to confirm these findings.

## Supplementary information

Supplementary Material

## Data Availability

The data sharing policy of Johnson & Johnson is available at https://innovativemedicine.jnj.com/our-innovation/clinical-trials/transparency. As noted on this site, requests for access to the study data can be submitted through Yale Open Data Access (YODA) Project site at http://yoda.yale.edu.
