# Supplementary material for: Daratumumab plus lenalidomide maintenance in newly diagnosed multiple myeloma after transplant: AURIGA subgroup analyses
Source: Blood Cancer J. 2025 Oct 6;15(1):154. doi: 10.1038/s41408-025-01355-0 (PMC12500855; doi:10.1038/s41408-025-01355-0)
Supplement: Supplementary file 1 — Supplementary Material [file 41408_2025_1355_MOESM1_ESM.docx]

# Supplementary Material

Daratumumab Plus Lenalidomide Maintenance in Newly Diagnosed Multiple Myeloma After Transplant: AURIGA Subgroup Analyses

Laahn Foster, Larry D. Anderson Jr, Alfred Chung, Chakra P. Chaulagain, Erin Pettijohn, Andrew J. Cowan, Caitlin Costello, Sarah Larson, Douglas W. Sborov, Kenneth H. Shain, Rebecca Silbermann, Peter Voorhees, Maria Krevvata, Huiling Pei, Sharmila Patel, Vipin Khare, Annelore Cortoos, Robin Carson, Thomas S. Lin, Ashraf Badros

# Listing of Institutional Review Boards/Independent Ethics Committees for AURIGA

## United States

Sterling Institutional Review Board, Atlanta, GA; Western Institutional Review Board, Puyallup, WA; University of California – San Diego Human Subjects Research Protection Program, La Jolla, CA; Ochsner Clinic Foundation, New Orleans, LA; University of California – Los Angeles Office of Human Research Protection Program, Los Angeles, CA; Wake Forest University Health Sciences Institutional Review Board, Winston-Salem, NC; Oregon Health & Science University Institutional Review Board, Portland, OR; Baylor Scott and White Institutional Review Board, Dallas, TX; University of Utah Institutional Review Board, Salt Lake City, UT; Cleveland Clinic Institutional Review Board, Cleveland, OH; New York University School of Medicine Institutional Review Board, New York, NY; Henry Ford Hospital Institutional Review Board, Detroit, MI; Columbia University Medical Center Institutional Review Board, New York, NY; Northside Hospital Institutional Review Board, Atlanta, GA; University of Miami Sylvester Comprehensive Cancer Center, Miami, FL; Biomedical Research Alliance of New York, Lake Success, NY; Mayo Clinic Institutional Review Board, Rochester, MN; Advarra, Columbia, MD; Greenville Health System Institutional Review Board, Greenville, SC; Reading Hospital Institutional Review Board, Reading, PA; University of Texas Southwestern Medical Center, Dallas, TX; Yale University Human Investigation Committee, New Haven, CT; University of Texas MD Anderson Cancer Center Institutional Review Board, Houston, TX; University of Iowa Institutional Review Board, Iowa City, IA; University of Kansas Medical Center Human Research Protection Program, Kansas City, KS; IntegReview Institutional Review Board, Austin, TX; University of Rochester School of Medicine Research Subjects Review Board, Rochester, NY; Dana Farber Cancer Institute Institutional Review Board, Boston, MA; Georgetown University Institutional Review Board, Washington, DC; University of Colorado Health Institutional Review Board, Fort Collins, CO; US Oncology Inc. Institutional Review Board, The Woodlands, TX; Human Studies Subcommittee (IRB#1) VA Puget South Health Care System, Seattle, WA; University of Miami Institutional Review Board Human Subjects Research Office, Miami, FL.

## Canada

UHN Research Ethics Board, Toronto, ON; MUHC Research Ethics Board, Montreal, QC; Comité d'Éthique du CHU de Québec-Université Laval, Québec, QC.

# Supplemental Table 1. AURIGA subgroup definitions

| **Subgroup category** | **Subgroup** | **Definition** |
| --- | --- | --- |
| Demographic and disease characteristics | ITT | Defined as all patients who were randomized to the study treatment (D-R or R) |
|  | Race | Patients who self-reported Black or White race at study screening |
|  | Age | Patients who were aged <65 years or ≥65 years at study screening |
|  | ISS disease stage | Patients with ISS stage I, II, or III disease at the time of MM diagnosis |
|  | Baseline response status | Patients who achieved a VGPR or ≥CR (CR/sCR) per IMWG 2016 criteria upon entering the study |
| Cytogenetic risk^a^ | Standard risk | Defined as any patient with 0 HRCAs per the original definition (per protocol) |
|  | High risk | Defined as any patient with ≥1 HRCAs per the original definition (per protocol), including del(17p), t(4;14), and/or t(14;16) |
|  | Revised standard risk | Defined as any patient with 0 HRCAs per the revised definition |
|  | Revised high risk | Defined as any patient with ≥1 HRCAs per the revised definition, including del(17p), t(4;14), t(14;16), t(14;20), and/or gain/amp(1q21) |
|  | 1 HRCA | Defined as any patient with 1 HRCA per the revised definition |
|  | ≥2 HRCAs | Defined as any patient with ≥2 HRCAs per the revised definition, also termed “ultra–high-risk” |
|  | Gain/amp(1q21)^b^ | Defined as any patient with gain/amp(1q21)^b^ irrespective of co-occurrence of other HRCAs |
|  | Isolated gain/amp(1q21)^b^ | Defined as any patient with gain/amp(1q21)^b^ without the presence of any other HRCA per the revised definition |
| Modified IMS 2024 high-risk MM^a,c^ | Modified IMS 2024 standard risk^c^ | A patient that was considered standard risk per modified IMS 2024 criteria if it was concluded with certainty that their cytogenetic results did not meet any of the 3 modified IMS 2024 high-risk subcategories listed below |
|  | Modified IMS 2024 high risk^c^ | Defined as any patient with ≥1 HRCAs per the modified IMS 2024 criteria, including ≥20% del(17p); del(1p32) co-occurring with gain/amp (1q21); or t(4;14), (14;16), and/or (14;20) co-occurring with gain/amp(1q21)^b^ and/or del(1p32) |
|  | ≥20% del(17p)^d^ | Any patient with the presence of del(17p)^d^ with a 20% threshold of positivity |
|  | t(4;14)/(14;16)/(14;20) + gain/amp(1q21) and/or del(1p32) | Any patient with an association of ≥2 of the following cytogenetic abnormalities: t(4;14) *and/or* t(14;16) *and/or* t(14;20) + gain/amp(1q21)^b^ *and/or* del(1p32) |
|  | Del(1p32) + gain/amp(1q21) | Any patient with the presence of del(1p32) co-occurring with any gain/amp 1q abnormalities (1q21)^b^ |

ITT, intent-to-treat; D-R, daratumumab/lenalidomide; R, lenalidomide; ISS, International Staging System; MM, multiple myeloma; VGPR, very good partial response; CR, complete response; sCR, stringent complete response; IMWG, International Myeloma Working Group; HRCA, high-risk cytogenetic abnormality; IMS, International Myeloma Society.

^a^Cytogenetic risk was assessed at diagnosis using available local fluorescence in situ hybridization/karyotype testing.

^b^Gain (3 copies) or amplification (≥4 copies) of 1q21.

^c^Per IMS 2024 criteria, high risk is determined by the presence of ≥1 of the following: high ß2M (>5.5 mg/dL) with creatinine (<1.2 mg/dL); ≥20% del(17p); TP53 mutation; biallelic del(1p32); monoallelic del(1p32) co-occurring with gain/amp(1q21); or t(4;14), t(14;16), and/or t(14;20) co-occurring with gain/amp(1q21) and/or monoallelic del(1p32). In this subgroup analysis of AURIGA, high risk per the IMS 2024 criteria was determined using IMS 2024 criteria for which data were available. In the AURIGA study, data were not collected (and thus not available) for TP53 mutations, beta-2-microglobulin levels and creatinine levels at the time of multiple myeloma diagnosis (ISS disease stage was gathered at diagnosis, but no associated creatinine levels were collected at baseline), and differentiation between monoallelic versus biallelic del(1p32).

^d^Determined by cancer clone fraction using analyses conducted on CD138-positive/purified cells.

# Supplemental Table 2. Demographic and disease characteristics (ITT population)

|  | **D-R**  **(N=99)** | **R**  **(N=101)** |
| --- | --- | --- |
| Age, n (%) |  |  |
| <65 years | 61 (61.6) | 61 (60.4) |
| ≥65 years | 38 (38.4) | 40 (39.6) |
| Race, n (%) |  |  |
| White | 67 (67.7) | 68 (67.3) |
| Black | 20 (20.2) | 24 (23.8) |
| ISS disease stage at diagnosis, n | 91 | 98 |
| I, n (%) | 40 (44.0) | 38 (38.8) |
| II, n (%) | 28 (30.8) | 37 (37.8) |
| III, n (%) | 23 (25.3) | 23 (23.5) |
| Patient response category at baseline,^a^ n (%) |  |  |
| VGPR | 71 (71.7) | 71 (70.3) |
| ≥CR | 28 (28.3) | 30 (29.7) |
| Cytogenetic risk at diagnosis per original definition,^b,c,d^ n | 92 | 89 |
| Standard risk, n (%) | 63 (68.5) | 66 (74.2) |
| High risk, n (%) | 22 (23.9) | 15 (16.9) |
| Unknown, n (%) | 7 (7.6) | 8 (9.0) |
| Cytogenetic risk at diagnosis per revised definition,^b,e^ n | 93 | 89 |
| Revised standard risk (0 HRCAs), n (%) | 52 (55.9) | 53 (59.6) |
| Revised high risk (≥1 HRCAs), n (%) | 32 (34.4) | 30 (33.7) |
| 1 HRCA | 21 (22.6) | 20 (22.5) |
| ≥2 HRCAs | 11 (11.8) | 10 (11.2) |
| Gain/amp(1q21) | 16 (17.2) | 22 (24.7) |
| Isolated gain/amp(1q21) | 10 (10.8) | 15 (16.9) |
| Unknown, n (%) | 9 (9.7) | 6 (6.7) |
| Cytogenetic risk per modified IMS 2024 criteria,^b,f^ n | 93 | 90 |
| Modified IMS 2024 standard risk, n (%) | 67 (72.0) | 68 (75.6) |
| Modified IMS 2024 high risk, n (%) | 17 (18.3) | 8 (8.9) |
| ≥20% del(17p) | 10 (10.8) | 2 (2.2) |
| t(4;14)/(14;16)/(14;20) + gain/amp(1q21) and/or del(1p32) | 5 (5.4) | 6 (6.7) |
| del(1p32) + gain/amp(1q21) | 4 (4.3) | 0 |
| Unknown, n (%) | 9 (9.7) | 14 (15.6) |

ITT, intent-to-treat; D-R, daratumumab/lenalidomide; R, lenalidomide; ISS, International Staging System; VGPR, very good partial response; CR, complete response, HRCA, high-risk cytogenetic abnormality; IMS, International Myeloma Society.

^a^Per International Myeloma Working Group 2016 criteria.

^b^Cytogenetic risk percent calculated based on the number of evaluable patients.

^c^High-risk cytogenetics per the original definition are defined as ≥1 abnormality including del(17p), t(4;14), and/or t(14;16).

^d^The imbalance in cytogenetic risk between arms, especially a higher number of patients with del(17p) for patients randomized to the D-R arm, was because some assessments were made on cytogenetic data at screening and some on cytogenetic data at the time of diagnosis.

^e^Revised high-risk cytogenetics per the revised definition are defined as ≥1 abnormality including del(17p), t(4;14), t(14;16), t(14;20), and/or gain/amp(1q21).

^f^High risk per the modified IMS 2024 criteria is defined as the presence of ≥20% del(17p); or the association of ≥2 of the following: t(4;14) or t(14;16) or t(14;20); gain/amp(1q21); or del(1p32). In the AURIGA study, data were not available on TP53 mutations, beta-2-microglobulin and creatinine levels at the time of multiple myeloma diagnosis, and differentiation between monoallelic versus biallelic del(1p32).

# Supplemental Figure 1. Subgroup analysis of MRD-negative (10^–5^) conversion rate overall.^a^


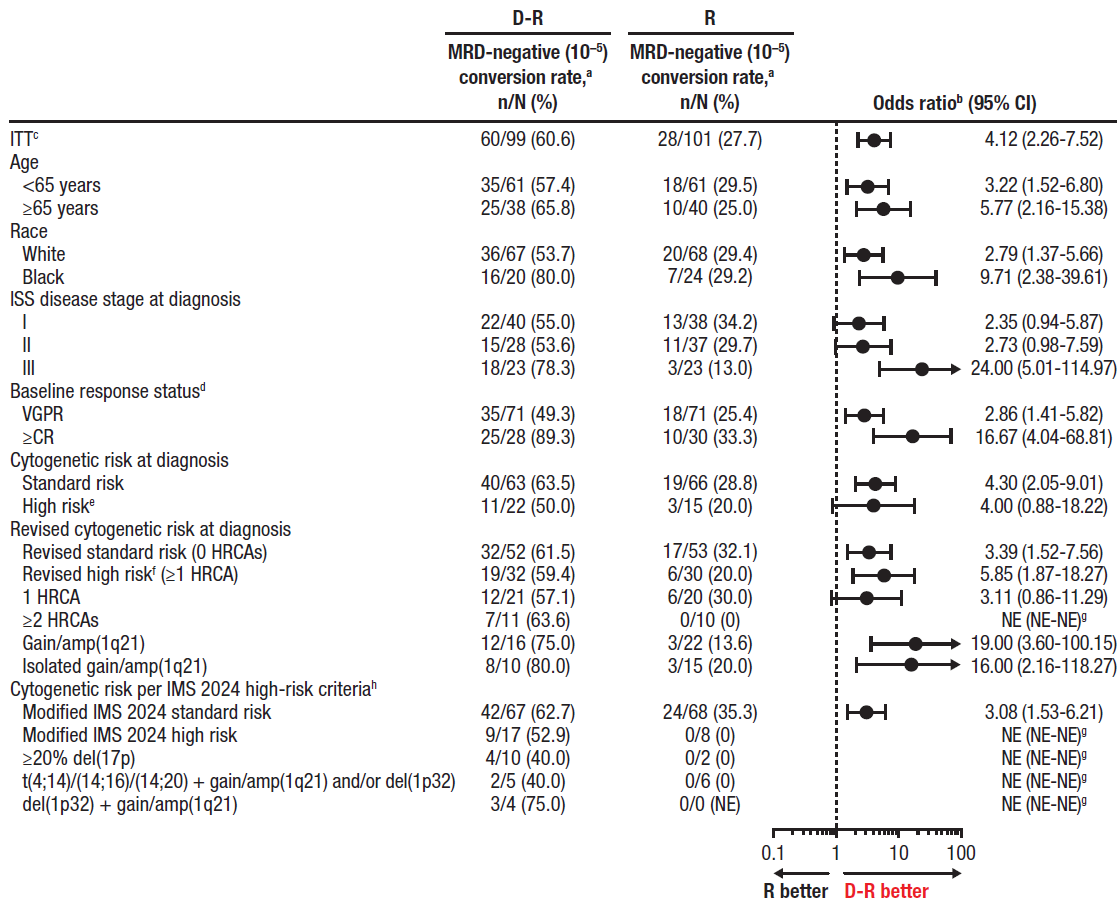


MRD, minimal residual disease; D-R, daratumumab/lenalidomide; R, lenalidomide; CI, confidence interval; ITT, intent-to-treat; ISS, International Staging System; VGPR, very good partial response; CR, complete response; HRCA, high-risk cytogenetic abnormality; NE, not estimable; IMS, International Myeloma Society.

^a^Defined as the proportion of patients who achieved MRD-negative status (by next-generation sequencing) any time after the date of randomization.

^b^Mantel-Haenszel estimate of the common odds ratio for stratified tables was used for ITT; Mantel-Haenszel estimate of the common odds ratio for unstratified tables was used for subgroups. An odds ratio >1 indicates an advantage for D-R maintenance.

^c^ITT population is defined as all patients who were randomized to treatment.

^d^Response status upon entering the study as assessed by International Myeloma Working Group 2016 criteria.

^e^High-risk cytogenetics per the original definition are defined as ≥1 abnormality including del(17p), t(4;14), and/or t(14;16).

^f^Revised high-risk cytogenetics per the revised definition are defined as ≥1 abnormality including del(17p), t(4;14), t(14;16), t(14;20), and/or gain/amp(1q21).

^g^Not evaluable because no patient in the R group had MRD-negative conversion.

^h^High risk per the modified IMS 2024 criteria is defined as the presence of ≥20% del(17p); del(1p32) co-occurring with gain/amp(1q21); or t(4;14), t(14;16), and/or t(14;20) co-occurring with gain/amp(1q21) and/or del(1p32). In the AURIGA study, data were not available on TP53 mutations, beta-2-microglobulin and creatinine levels at the time of multiple myeloma diagnosis, and differentiation between monoallelic versus biallelic del(1p32).

# Supplemental Figure 2. Subgroup analysis of ≥CR rate (best response on study).^a^


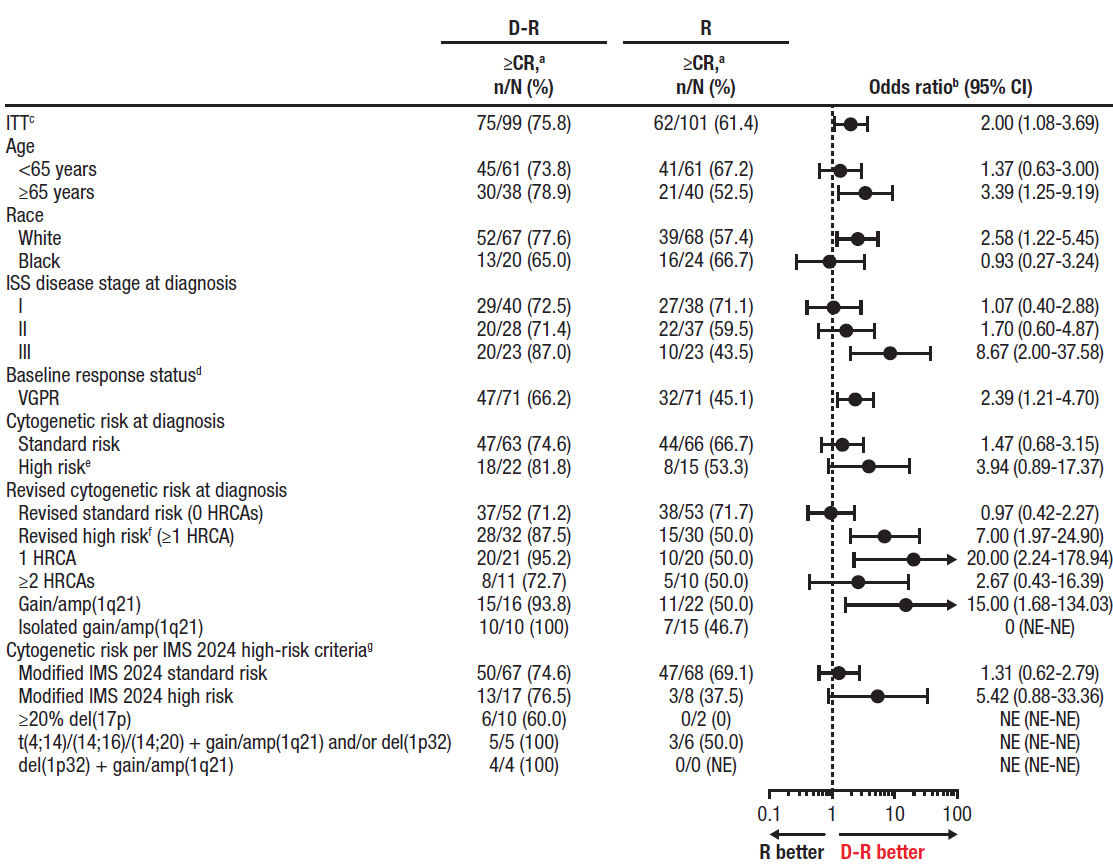


CR, complete response; D-R, daratumumab/lenalidomide; R, lenalidomide; CI, confidence interval; ITT, intent-to-treat; ISS, International System Staging; VGPR, very good partial response; HRCA, high-risk cytogenetic abnormality; NE, not estimable; IMS, International Myeloma Society.

^a^Achievement of ≥CR rate during the treatment period (best response on study).

^b^Mantel-Haenszel estimate of the common odds ratio for stratified tables was used for ITT; Mantel-Haenszel estimate of the common odds ratio for unstratified tables was used for subgroups. An odds ratio >1 indicates an advantage for D-R maintenance.

^c^ITT population is defined as all patients who were randomized to treatment.

^d^Response status upon entering the study per as assessed by International Myeloma Working Group 2016 criteria.

^e^High-risk cytogenetics per the original definition are defined as ≥1 abnormality including del(17p), t(4;14), and/or t(14;16).

^f^Revised high-risk cytogenetics per the revised definition are defined as ≥1 abnormality including del(17p), t(4;14), t(14;16), t(14;20), and/or gain/amp(1q21).

^g^High risk per the modified IMS 2024 criteria is defined as the presence of ≥20% del(17p); del(1p32) co-occurring with gain/amp(1q21); or t(4;14), t(14;16), and/or t(14;20) co-occurring with gain/amp(1q21) and/or del(1p32). In the AURIGA study, data were not available on TP53 mutations, beta-2-microglobulin and creatinine levels at the time of multiple myeloma diagnosis, and differentiation between monoallelic versus biallelic del(1p32).

# Supplemental Figure 3. PFS analysis of all subgroups.


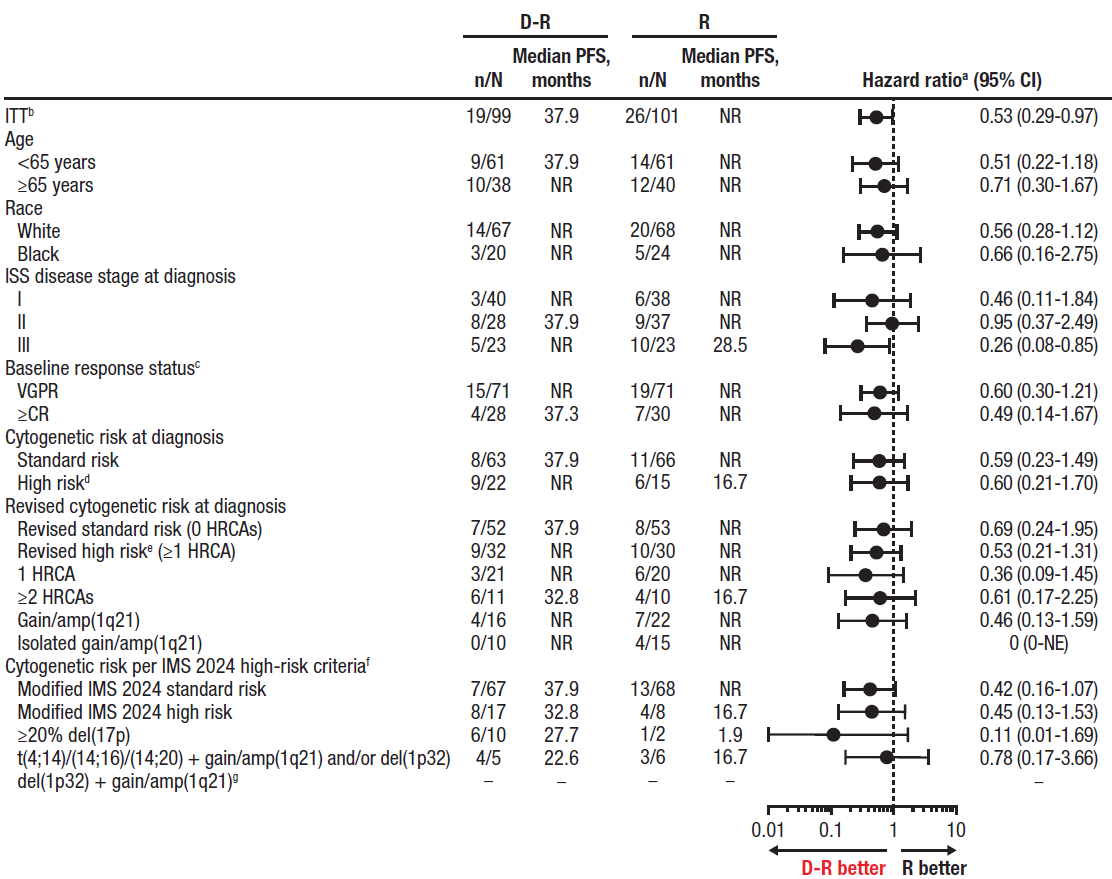


PFS, progression-free survival; D-R, daratumumab/lenalidomide; R, lenalidomide; CI, confidence interval; ITT, intent-to-treat; NR, not reached; ISS, International Staging System; VGPR, very good partial response; CR, complete response; HRCA, high-risk cytogenetic abnormality; NE, not estimable; IMS, International Myeloma Society.

^a^Hazard ratio and 95% CI from a Cox proportional hazards model with treatment as the sole explanatory variable. A hazard ratio <1 indicates an advantage for D-R maintenance.

^b^ITT population is defined as all patients who were randomized to treatment.

^c^Response status upon entering the study per as assessed by International Myeloma Working Group 2016 criteria.

^d^High-risk cytogenetics per the original definition are defined as ≥1 abnormality including del(17p), t(4;14), and/or t(14;16).

^e^Revised high-risk cytogenetics per the revised definition are defined as ≥1 abnormality including del(17p), t(4;14), t(14;16), t(14;20), and/or gain/amp(1q21).

^f^High risk per the modified IMS 2024 criteria is defined as the presence of ≥20% del(17p); del(1p32) co-occurring with gain/amp(1q21); or t(4;14), t(14;16), and/or t(14;20) co-occurring with gain/amp(1q21) and/or del(1p32). In the AURIGA study, data were not available on TP53 mutations, beta-2-microglobulin and creatinine levels at the time of multiple myeloma diagnosis, and differentiation between monoallelic versus biallelic del(1p32).

^g^This high-risk subcategory (del[1p32] co-occurring with gain/amp[1q21]) was excluded from this PFS analysis graph as there were no patients in R group that fell in this subcategory. Of the 4 patients in D-R group who were in this high-risk subcategory, all were censored; median PFS (95% CI) was NR (NE, NE).

# Supplemental Figure 4. Subgroup analysis of PFS by A) gain/amp(1q21),^a^ B) isolated gain/amp(1q21),^b^ and C) response at study entry.^c^


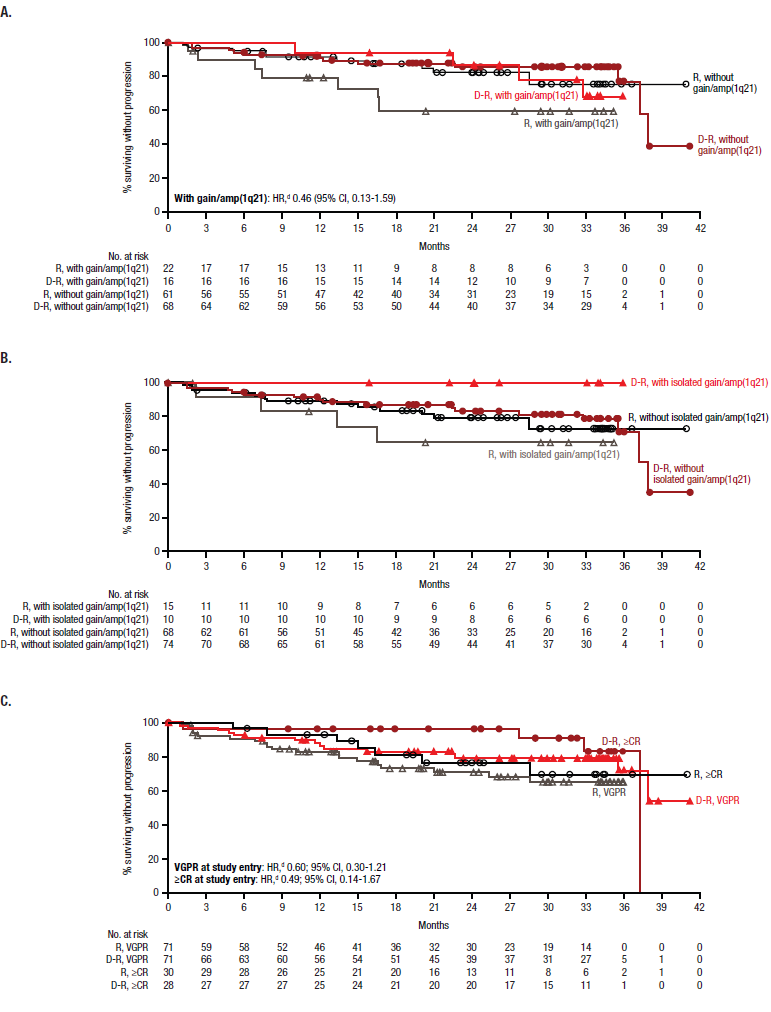


PFS, progression-free survival; D-R, daratumumab/lenalidomide; R, lenalidomide; HR, hazard ratio; CI, confidence interval; CR, complete response; VGPR, very good partial response.

^a^With (presence of) gain/amp(1q21) refers to gain/amp(1q21) abnormality regardless of status of the other HRCAs per the revised definition. Without gain/amp(1q21) refers to revised high risk or revised standard risk without gain/amp(1q21) abnormality.

^b^With (presence of) isolated gain/amp(1q21) refers to gain/amp(1q21) abnormality without the other HRCAs (del[17p], t[4;14], t[14;16], or t[14;20]). Without isolated gain/amp(1q21) refers to revised high risk or revised standard risk without isolated gain/amp(1q21) abnormality.

^c^Response at study entry per International Myeloma Working Group 2016 criteria.

^d^HR and 95% CI from a Cox proportional hazards model with treatment as the sole explanatory variable. An HR <1 indicates an advantage for D-R maintenance.
